# Interruptions in Supplies of Second-Line Antituberculosis Drugs — United States, 2005–2012

**Published:** 2013-01-18

**Authors:** Barbara J. Seaworth, Kim Field, Jennifer Flood, Jouhayna Saliba, Sundari R. Mase, Ann Cronin, Neha Shah, John Jereb, Terence Chorba

**Affiliations:** Univ of Texas Health Science Center; San Diego County Tuberculosis Control Program; California Dept of Public Health; Center for Drug Evaluation and Research, Food and Drug Administration; Div of Tuberculosis Elimination, National Center for HIV, Viral Hepatitis, STD, and TB Prevention, CDC

Second-line drugs (SLDs) are essential for treating multidrug-resistant and extensively drug-resistant tuberculosis (MDR TB* and XDR TB†). Drug shortages, in which supplies of all clinically interchangeable versions of a given Food and Drug Administration (FDA)–regulated drug become inadequate to meet actual or projected user demand, have been well-documented in many areas of medicine; for several years, drug shortages in the United States have affected the availability of SLDs for treatment of TB. In November 2010, a nationwide survey of TB control programs conducted by the National Tuberculosis Controllers Association (NTCA) indicated that shortages and other problems that hinder access to SLDs interfere with patient care and could promote the development of drug resistance as well as the transmission of drug-resistant *Mycobacterium tuberculosis*. This report focuses on the growing issue of TB drug shortages and summarizes the findings of that survey, which indicated that 26 (79%) of the 33 responding health departments, representing approximately 75% of the U.S. TB burden, reported MDR TB during 2005–2010. Of these 26, 21 (81%) faced difficulties with SLD procurement, citing nationwide shortages (100%), shipping delays (71%), lack of resources (62%), and a complicated procurement process related to investigational new drug (IND) protocols (48%) as the main reasons. Adverse outcomes or other problems related to difficulties with SLD procurement were reported by 19 (90%) of 21 jurisdictions, with treatment delay (58%), a treatment lapse or interruption (32%), or the use of an inadequate regimen (32%) most commonly reported. Potential solutions for alleviating SLD shortages include stockpiling drugs centrally, sharing SLDs among jurisdictions, obtaining drugs from foreign manufacturers, and taking advantage of new legal requirements for drug suppliers to report shortages and impending shortages to FDA within a specified timeframe. Reliable, consistent access to SLDs will require the collaboration of CDC, FDA, state and local health departments, national health professional societies, and the pharmaceutical industry.

TB is caused by the bacterium *M. tuberculosis*, which is most typically transmitted through the air from one person to another. For most TB cases, cure is achieved with a standard combination of drugs. For the treatment of confirmed or suspected TB disease, isoniazid, rifampin, pyrazinamide, and ethambutol are the four first-line drugs used worldwide as a 6-month standard regimen. In contrast, MDR TB generally requires 18–24 months of treatment with five or six drugs that are less effective, more toxic, and more costly than first-line drugs. As a result, MDR TB causes greater morbidity, and, overall, patient outcomes are worse.

Currently, CDC and FDA are collaborating to identify solutions to ameliorate a national shortage of isoniazid (1). SLD shortages also can disrupt treatment of drug-susceptible TB in patients who cannot tolerate first-line drugs and can complicate the treatment of MDR TB and XDR TB, putting patients and communities at greater risk for morbidity and mortality. For example, in April 2011, shortages of capreomycin and amikacin, two SLDs used to treat MDR TB and XDR TB, posed a serious threat for a father and his infant who had MDR TB. Despite intensive efforts by public health personnel to obtain the two drugs, the initiation of treatment was delayed by 8 days for both patients, prolonging the father’s infectious period and thereby increasing the risk for transmission to the community. The infant, who had basilar meningitis and severe communicating hydrocephalus, was placed in a particularly dangerous situation. TB meningitis in young children is a medical emergency, and delays in treatment lead to worse outcomes (2), such as severe cognitive impairment, epilepsy, and death (3).

In November 2010, NTCA, an organization of state, local, and territorial health officials, surveyed its membership in 50 states, 10 large cities, five territories, and the three freely-associated Pacific Island nations. An NTCA web-based questionnaire addressed medication procurement issues, costs, and treatment delays related to interruptions in SLD access. The survey was sent to NTCA members who had a functioning e-mail address (61 [90%] of 68 members), and those surveyed had 1 month to respond to the questionnaire.

Of the 61 surveyed jurisdictions, 33 (54%) responded. Of those jurisdictions that responded, 29 (88%) represented state TB programs, four (12%) represented large cities, and 26 (79%) reported having had an MDR TB case in the past 5 years. Of these 26, 21 (81%) stated that their program had faced challenges obtaining MDR TB drugs in the past 5 years (Table), citing reasons such as nationwide shortages (21 [100%]), shipping delays (15 [71%]), lack of resources to pay for SLDs (presumably meaning that neither the program, the patient, nor a health insurer could afford the drugs) (13 [62%]), and a complicated procurement process related to IND protocols (10 [48%]). Of the 21 jurisdictions reporting challenges with drug procurement, 19 reported adverse outcomes or other problems, such as treatment delay (11 [58%] of 19), a treatment lapse or interruption (six [32%] of 19), the use of an inadequate regimen (six [32%] of 19), and substantial staff time expended for drug procurement (13 [68%] of 19).

## Editorial Note

In the United States in recent years, interruptions in access to SLDs have hindered MDR TB and XDR TB treatment. CDC does not formally monitor SLD supplies, but TB control officials in local and state health departments request assistance from CDC when they encounter difficulties with drug procurement. Since 2005, CDC has received reports of difficulty obtaining each of the following SLDs: streptomycin, cycloserine, ethionamide, rifabutin, amikacin, capreomycin, and kanamycin. Shortages of rifampin also have been reported, and, in the past 2 months, a national shortage of isoniazid has developed (1). Shortages of MDR TB medications have been experienced by most U.S. TB programs that have diagnosed and reported MDR TB cases. In 2010, most (130 [73%] of 178) of these reports of SLD shortages involved sterile injectable antibiotics essential for the treatment of MDR TB. Since September 2011, the availability of injectable SLDs for MDR TB treatment has been precarious. Kanamycin is no longer produced in the United States, streptomycin has been intermittently unavailable because of increased international demand, and capreomycin and amikacin have been available on an intermittent basis in only small amounts because of manufacturing problems and lack of raw materials.

Drug shortages in the Unites States have become increasingly common. In 2005, a total of 61 impending drug shortages were reported to FDA; in 2010, there were 178. FDA maintains a website to alert the public of drug shortages and their causes (4). A recent FDA review of medical product shortages underscored the complexity of the problem, identifying poor drug quality leading to product recalls as the most common cause for a shortage. Difficulties procuring raw materials and components also were problems. Early notification to FDA can help prevent some impending shortages. For example, in 2010, of 178 drugs with impending drug shortages, FDA was able to prevent shortages of 38 (21%); in 2011, 195 shortages were prevented (5,6), and, as of November 1, 2012, shortages of at least 150 drugs had been prevented in 2012 (Jouhayna Saliba, PharmD, Center for Drug Evaluation and Research, FDA; personal communication; 2013).

As with any drug shortage, SLD shortages can contribute to adverse outcomes such as delays and interruptions in treatment, the need to use potentially less effective treatment regimens, prolonged infectiousness and increased transmission of drug-resistant TB in the community, the further development of drug resistance, and worse outcomes for patients (7). Additionally, drug shortages lead to rationing, increased drug costs, and inefficient use of staff time, and increase the risk for medication errors because regimens must be adjusted, leading to confusion over drug administration schedules, adverse reactions, and interactions (8).

In March 2011, the Federal Advisory Council for the Elimination of Tuberculosis, which provides recommendations for TB elimination to the U.S. Department of Health and Human Services, formed a workgroup to design strategies for improving SLD access. In November 2011, a recurrent bimonthly national forum of 60 TB experts (including members of the Federal Advisory Council for the Elimination of Tuberculosis, state TB controllers, members of the CDC-funded Regional Training and Medical Consultation Centers, patient advocacy groups, and personnel from CDC and FDA), convened by National Jewish Health to discuss clinical and programmatic issues specific to MDR TB and XDR TB, assessed the findings of the NTCA survey and data from FDA and CDC in the context of case presentations. Potential solutions for improving continuity of SLD supplies were suggested by the TB experts present, although no report resulted from the meeting; these proposed solutions included the sharing of drugs in short supply among state and local TB programs, centralized drug stockpiling, obtaining drugs from foreign manufacturers when not available in the United States, and having CDC be responsible for a nationally centralized IND application protocol for certain drugs to expedite access to these drugs for all U.S. patients. Currently, CDC is responsible for nine IND protocols and is developing such a proposal for clofazimine, an SLD.

What is already known on this topic?

Drug shortages, defined as situations in which the total supply of all clinically interchangeable versions of a given Food and Drug Administration (FDA)–regulated drug is inadequate to meet the current or projected demand at the user level, are a well-documented problem in many areas of medicine.

What is added by this report?

A nationwide survey of U.S. tuberculosis (TB) control programs showed that 81% of responding jurisdictions that reported having a multidrug-resistant TB case have experienced shortages of second-line drugs (SLDs) for TB treatment and that these shortages have resulted in adverse outcomes for programs and patients. The main reasons cited include nationwide shortage (100%), shipping delays (71%), lack of resources (62%), and a complicated procurement process related to investigational new drug protocols (48%). Shortages of SLDs have contributed to delays (58%) and interruptions in treatment (32%) and the need to use potentially less effective treatment regimens (32%), and can prolong infectiousness, increase transmission of drug-resistant TB in the community, further the development of acquired drug resistance, and lead to worse outcomes for patients.

What are the implications for public health practice?

Potential solutions for improving continuity of SLD supplies include the sharing of drugs in short supply among state and local TB programs, centralized drug stockpiling, obtaining drugs from foreign manufacturers when not available in the United States, and having CDC be responsible for a nationally centralized investigational new drug application protocol for certain drugs to expedite access to these drugs for all U.S. patients. On July 9, 2012, the FDA Safety and Innovation Act of 2012 was signed into law. In the law, Congress provided FDA with authorities to combat shortages of drug products in the United States and imposed requirements on manufacturers regarding early notification to FDA of issues that could lead to a potential shortage or disruption in supply of a product.

The findings in this report are subject to at least one limitation. Because of the low response rate (54%) to the NTCA survey, the results might not be representative of the nation’s experience with SLD shortages generally.

On July 9, 2012, the FDA Safety and Innovation Act of 2012 was signed into law. In the law, Congress provided FDA with authorities to combat shortages of drug products in the United States and imposed requirements on manufacturers regarding early notification to FDA of issues that could lead to a potential shortage or disruption in supply of a product.

In October 2011, Executive Order 13588 directed FDA and the U.S. Department of Justice to take action to combat drug shortages, protect consumers, and prevent deliberate price inflation (9). Maintaining access to SLDs will require the collaboration of CDC (because of its role as the central component of the national TB program), FDA, and other partners, including the Global Drug Facility, the Treatment Action Group, local and state health departments, national and international health professional societies, and the pharmaceutical industry. To report information about shortages or supply issues, manufacturers can send updates by e-mail to drugshortages@fda.hhs.gov. State and local health departments, health-care professionals, and patients also are encouraged to notify FDA of shortages, using the same e-mail address.

## Figures and Tables

**TABLE:** Number and percentage of local, state, and territorial health departments experiencing challenges in obtaining second-line drugs (SLDs)* for tuberculosis treatment in the past 5 years, by selected characteristics — National Tuberculosis Controllers Association member survey, United States, 2010

Characteristic	No.	(%)
**Faced any challenges obtaining SLDs in the past 5 years**	**21/33**	**(64)**
Nationwide shortages	21/21	(100)
Shipping delays	15/21	(71)
Medications too expensive for their program	13/21	(62)
Medications too expensive for uninsured	10/21	(48)
Delays caused by IND protocol submission	10/21	(48)
Medications too expensive for insured patients	8/21	(38)
Payer bureaucracy	7/21	(33)
**Adverse effects and other problems**		
Substantial staff time diverted by drug procurement	13/19	(68)
Delay in starting treatment	11/19	(58)
Treatment lapse and interruption	6/19	(32)
Inadequate regimen	6/19	(32)

**Abbreviation:** IND = investigational new drug.

* Including capreomycin, kanamycin, amikacin, moxifloxacin, levofloxacin, para-aminosalicylate sodium, cycloserine, ethionamide, linezolid, and clofazimine.

## Footnotes

* MDR TB is caused by *Mycobacterium tuberculosis* that is resistant to at least isoniazid and rifampin, the two most potent of the four first-line anti-TB drugs. Ethambutol and pyrazinamide are the other two first-line TB drugs.

† XDR TB is MDR TB with additional resistance to any fluoroquinolone and at least one of the injectable TB drugs (kanamycin, capreomycin, and amikacin).

## References

[1] CDC (2012). National shortage of isoniazid 300 mg tablets. MMWR.

[2] Sheu JJ, Yuan RY, Yang CC (2009). Predictors for outcome and treatment delay in patients with tuberculous meningitis. Amer J Medical Sci.

[3] Anderson NE, Somaratne J, Mason DF, Holland D, Thomas MG (2010). Neurological and systemic complications of tuberculous meningitis and its treatment at Auckland City Hospital, New Zealand. J Clin Neurosci.

[4] Food and Drug Administration (2011). FDA working to lessen patient impact from drug shortages.

[5] Food and Drug Administration (2012). A review of FDA’s approach to medical product shortages, 2011.

[6] Office of Science and Data Policy, Office of the Assistant Secretary for Planning and Evaluation, US Department for Health and Human Services (2011). Economic analysis of the causes of drug shortages, October 2011.

[7] Leimane V, Dravniece G, Riekstina V (2010). Treatment outcome of multidrug/extensively drug-resistant tuberculosis in Latvia, 2000–2004. Eur Respir J.

[8] De Oliveira GS, Theilken LS, McCarthy RJ (2011). Shortage of perioperative drugs: implications for anesthesia practice and patient safety. Anesth Analg.

[9] Office of the Press Secretary, White House (2011). Fact sheet: Obama administration takes action to reduce prescription drug shortages in the U.S..

